# Screening of oxidative stress-related signature genes and construction of an interpretable discriminant model for preeclampsia

**DOI:** 10.3389/fgene.2026.1855189

**Published:** 2026-10-29

**Authors:** Leilei Tian, Xuyuan Ma, Yu Zhang, Tingting Hao, Hanxue Wang, Haohao Meng, Hong Xin

**Affiliations:** 1 Department of Graduate School, Hebei Medical University, Shijiazhuang, China; 2 Department of Obstetrics and Gynecology, The Second Hospital of Hebei Medical University, Shijiazhuang, Hebei, China

**Keywords:** bioinformatics, interpretable discriminant model, machine learning, preeclampsia, signature genes

## Abstract

**Background:**

Preeclampsia (PE) is a serious pregnancy complication threatening maternal and fetal health, and its onset and progression are closely associated with placental oxidative stress. However, a systematic understanding of oxidative stress-related genes in PE and their roles in immune regulation remains lacking.

**Objective:**

To identify oxidative stress-related signature genes in preeclampsia, validate their expression and tissue localization in placental tissues, construct an interpretable machine learning discriminant model, and explore their relationship with placental immune infiltration.

**Methods:**

Nine GEO datasets were classified into three groups: a feature gene screening group (GSE10588, GSE75010), an artificial neural network training group (GSE30186, GSE44711, GSE54618, GSE24129, GSE43942, GSE147776), and an independent validation group (GSE25906). Differentially expressed genes (DEGs) were identified by limma, and their intersection with oxidative stress-related genes yielded candidate DEOSRGs. Multi-algorithm recursive feature elimination (SVM-RFE, RF-RFE, XGB-RFE) with five-fold cross-validation selected the core signature genes. Placental protein expression and trophoblast localization were assessed in clinical specimens (50 PE vs. 50 controls). An artificial neural network (ANN) discriminant model was constructed on the training group, with GSE25906 used for independent validation and SHAP-based interpretability analysis. Immune infiltration was analyzed by CIBERSORT on GSE75010.

**Results:**

We identified 87 DEGs (65 upregulated, 22 downregulated) and 17 candidate DEOSRGs. Consensus recursive feature elimination screening yielded five core signature genes: LEP, HK2, NDRG1, membrane metallo-endopeptidase, and SERPINA3. Western blotting confirmed significant upregulation of all five proteins in PE placental tissues (P < 0.05), and immunohistochemistry localized positive signals predominantly to placental villous syncytiotrophoblasts. The ANN model achieved a 5-fold cross-validation AUC of 0.888 ± 0.010 and maintained 0.819 ± 0.010 in GSE25906, with LEP contributing most to classification. Immune infiltration profiling showed these genes were closely correlated with alterations in monocytes, M2 macrophages, Tregs, and neutrophils.

**Conclusion:**

Five oxidative stress-related signature genes (LEP, HK2, NDRG1, MME, and SERPINA3) were identified and found significantly upregulated in PE placental tissues. An interpretable neural network discriminant model was constructed and independently validated. These biomarkers reflect placental pathophysiological alterations and may contribute to PE progression through coordinated modulation of oxidative stress and the maternal–fetal immune microenvironment.

## 1 Introduction

Preeclampsia (PE), a major subtype of hypertensive disorders of pregnancy, remains one of the leading causes of maternal and fetal morbidity and mortality worldwide, with a global incidence of approximately 2%–8% (Lyu et al., 2021; Duley, 2009). Clinically, PE is characterized by new-onset hypertension after 20 weeks of gestation, accompanied by proteinuria and multiple organ dysfunction. The kidneys and liver are among the most commonly affected organs. Without timely intervention, PE may progress to eclampsia, HELLP syndrome (hemolysis, elevated liver enzymes, and low platelet count), or even multiple organ failure, thereby posing a serious threat to maternal and neonatal health (Sibai, 2003).

Current evidence suggests that the pathogenesis of PE originates from pathological alterations during placental development, primarily including inadequate trophoblast invasion and impaired remodeling of the uterine spiral arteries, which ultimately result in reduced placental perfusion and a hypoxic–ischemic state (Wikström et al., 2009; Taravati and Tohidi, 2018; Khodzhaeva et al., 2016; Lyall, 2006). Placental ischemia can further trigger local ischemia–reperfusion injury, leading to excessive production of reactive oxygen species (ROS) and the establishment of oxidative stress (Roberts and Gammill, 2005). This process constitutes a central event in the pathophysiology of PE: excessive ROS not only directly damages placental tissues but also activates systemic inflammatory cascades, disrupts vascular endothelial function, and promotes the progression of the disease from a localized placental disorder to a systemic syndrome (Seki, 2017; Walsh, 1998). According to the classical “two-stage theory,” the first stage involves inadequate placental perfusion that induces hypoxia and oxidative stress, whereas the second stage is characterized by the release of placental factors that cause widespread maternal endothelial dysfunction and systemic clinical manifestations. In this framework, oxidative stress serves as a critical “signal amplifier” linking these two stages (Myatt and Cui, 2004).

As a placenta-derived disorder, PE is also closely associated with immune tolerance at the maternal–fetal interface. Increasing evidence has shown that immune tolerance is disrupted in patients with PE, resulting in an imbalance between pro-inflammatory and anti-inflammatory signaling, which in turn contributes to maternal systemic endothelial dysfunction and clinical manifestations (Saito et al., 2007). Therefore, a systematic evaluation of alterations in the maternal systemic immune status and the local placental immune microenvironment is of great importance for a deeper understanding of the pathogenesis of PE.

In recent years, the rapid development of bioinformatics and artificial intelligence has made it possible to identify key disease-associated biomarkers through high-throughput data mining. Artificial neural networks (ANNs), computational models inspired by the structure of biological neural systems, are designed to iteratively optimize network parameters through large-scale sample training, thereby enabling the fitting and recognition of complex data patterns (Schmidhuber, 2015) Owing to their capacity to capture nonlinear interactions among variables, ANNs have shown considerable promise in disease classification, risk prediction, and biomarker identification (Vadapalli et al., 2022; Libbrecht and Noble, 2015; Esteva et al., 2017). In complex disorders such as PE, which involve coordinated regulation by multiple genes and pathways, ANNs are capable of uncovering latent patterns in gene expression data without predefining interaction structures, thus providing a novel technical framework for systematically elucidating disease mechanisms.

Despite their strong nonlinear modeling capability, complex machine learning models such as ANNs often lack transparency in their internal decision-making processes and are therefore considered typical “black-box” models. In biomedical research, it is essential to further interpret the direction and magnitude of the contribution of each input feature to the prediction outcome, so as to enhance the reliability of model outputs and facilitate biological interpretation. SHAP (SHapley Additive exPlanations) is a *post hoc* interpretation method based on Shapley values from cooperative game theory. It decomposes the prediction for an individual sample into the sum of a baseline value and the contributions of each feature, thereby enabling both local and global interpretation within a unified additive explanatory framework (Lundberg and Lee, 2017). In recent years, SHAP has been increasingly applied in bioinformatics and omics studies to interpret key feature contributions in tasks such as disease classification, tumor subtype identification, and biological sequence prediction.

Based on the above considerations, the present study aimed to integrate publicly available transcriptomic datasets with clinical placental samples to systematically identify key genes closely associated with oxidative stress in PE. Furthermore, an ANN discriminant model based on these signature genes was constructed and validated across multiple independent cohorts, and SHAP was employed to interpret the key decision-driving features of the model. In addition, the potential relationship between the identified signature genes and the placental immune microenvironment was characterized. This study provides new insights into the molecular alterations underlying PE and establishes a candidate biomarker panel for disease characterization. The comprehensive technical workflow of this study is depicted in Figure 1.

**FIGURE 1:**
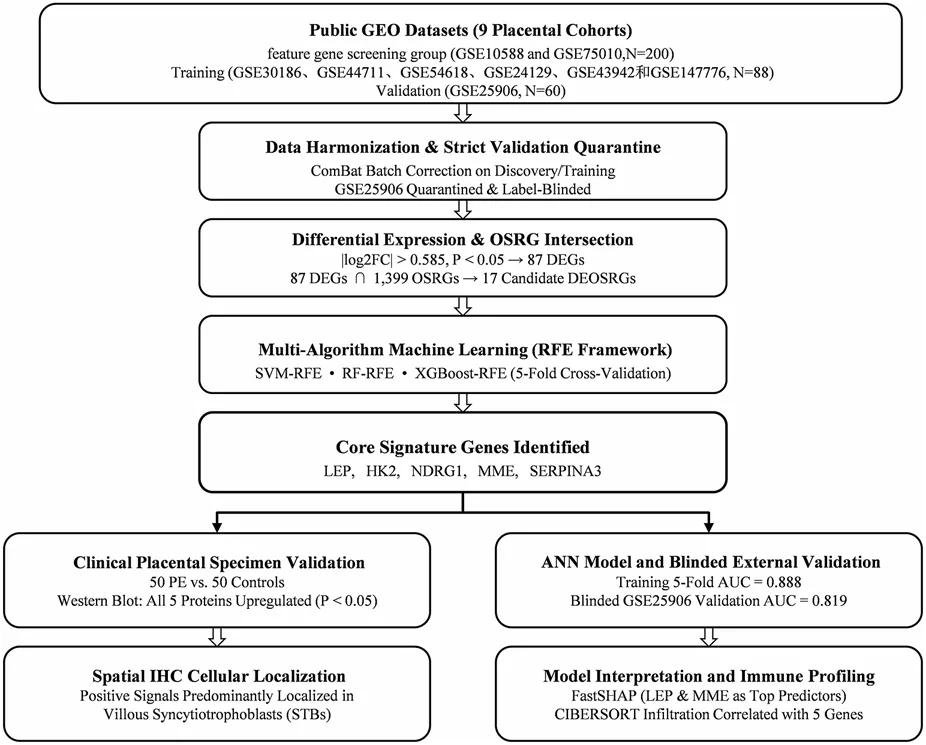
Study Design and Methodological Workflow. Schematic diagram illustrating multi-cohort data isolation, consensus biomarker selection, clinical placental validation, and explainable neural network modeling.

## 2 Materials and methods

### 2.1 Data acquisition and preprocessing

A total of nine GEO datasets were included in this study. GSE10588 and GSE75010 were assigned to the feature gene screening group for differential expression analysis and signature gene identification. GSE30186, GSE44711, GSE54618, GSE24129, GSE43942, and GSE147776 were used as the ANN training group for model construction. GSE25906 served as an independent validation cohort for verifying the differential expression of the five signature genes and evaluating the performance of the ANN model. Specifically, GSE30186, GSE44711, and GSE54618 were based on the GPL10558 platform; GSE24129 was based on GPL6244; GSE43942 was based on GPL10191; GSE147776 was based on GPL20844; and the independent validation cohort GSE25906 was based on GPL6102. According to the platform annotation files corresponding to each dataset, probe IDs were first mapped to gene symbols. For cases in which multiple probes corresponded to the same gene, the median expression value was used for probe collapsing. Empirical scale harmonization was performed based on the numerical distribution of each dataset: cohorts deposited with normalized log2 values—including GSE75010, GSE24129, GSE147776, GSE54618 and the external validation cohort GSE25906—were maintained in their native scale; cohorts deposited as un-logged linear signal intensities (GSE10588, GSE43942, GSE30186, and GSE44711) were explicitly converted using log2 (x + 1) or shifted log2 transformation prior to probe mapping. Batch effects across the training cohorts were adjusted using the ComBat method from the sva package in R in an unsupervised manner without incorporating disease outcome labels into the model. The independent validation dataset GSE25906 was processed separately and remained completely quarantined during feature selection and model training. The effectiveness of batch correction in the training sets was confirmed by principal component analysis (PCA).

### 2.2 Identification of differentially expressed genes (DEGs)

Differential expression analysis was performed on the feature gene screening group (GSE10588 and GSE75010) using the limma package in R. The screening criteria were set as Benjamini–Hochberg adjusted P < 0.05 and absolute log fold change (|logFC|) > 0.585. The identified DEGs were visualized using volcano plots and heatmaps generated with the ggplot2 and pheatmap packages.

### 2.3 Functional enrichment and pathway analysis of DEGs

To explore the potential biological functions and signaling pathways associated with the DEGs, Gene Ontology (GO) enrichment analysis was conducted using the clusterProfiler package in R. The GO analysis included three categories: biological process (BP), cellular component (CC), and molecular function (MF). Enrichment results with P < 0.05 were considered statistically significant and were visualized using the ggplot2 package.

### 2.4 Identification of DEOSRGs and construction of the PPI network

Differentially expressed oxidative stress-related genes (DEOSRGs) were obtained by intersecting the DEGs with a predefined set of oxidative stress-related genes (OSRGs). The predefined OSRGs list was derived from the Supplementary Material of a previously published study (Wang et al., 2022). It obtained from the GeneCards database using “oxidative stress” as the search term. Genes with a relevance score ≥ 7 were retained, resulting in 1,399 OSRGs for subsequent analysis. The DEOSRGs were subsequently uploaded to the STRING database to construct a protein-protein interaction (PPI) network. Network visualization was performed using the igraph package in R, and chromosomal localization was illustrated using Rcircos.

### 2.5 Machine learning-based screening of signature genes

To identify the most discriminative features, three recursive feature elimination (RFE) approaches—support vector machine-recursive feature elimination (SVM-RFE), random forest-recursive feature elimination (RF-RFE), and extreme gradient boosting-recursive feature elimination (XGB-RFE)—were applied for feature selection and importance ranking. All analyses were conducted in R using the caret package. Model construction was organized within a stratified five-fold cross-validation framework, where within each outer training split (four folds), an inner cross-validation loop was implemented to perform grid-search hyperparameter optimization (cost C and kernel parameter gamma for SVM; mtry and ntree for RF; max_depth and learning_rate for XGBoost), and the held-out outer fold was reserved exclusively for unbiased performance estimation. The independent validation cohort (GSE25906) was strictly held out and never accessed during CV, RFE, or parameter tuning. Optimal hyperparameters were selected based on maximizing ROC-AUC across inner folds. During the RFE procedure, features were iteratively removed from 17 down to 1 feature, and AUC-versus-feature-number trajectories as well as marginal performance change (ΔAUC) were analyzed to determine the optimal gene combination. The top 7 feature subsets from each algorithm were extracted, and their consensus intersection was defined as the core signature panel.

### 2.6 Tissue validation

#### 2.6.1 Sample source

Placental tissue samples were collected from 50 pregnant women with PE and 50 pregnant women with normal pregnancies who delivered at the Second Hospital of Hebei Medical University between October 2023 and January 2025.

The inclusion criteria for the PE group were based on the Chinese Guidelines for the Diagnosis and Treatment of Hypertensive Disorders in Pregnancy (2020): new-onset hypertension after 20 weeks of gestation (blood pressure ≥ 140/90 mmHg) accompanied by any one of the following: 24-h urinary protein ≥ 0.3 g, urine protein/creatinine ratio ≥ 0.3, or random urine protein ≥(+); or, in the absence of proteinuria, abnormal changes in major organs or systems such as the heart, lungs, liver, or kidneys. All included participants had singleton pregnancies, gestational age confirmed by first-trimester ultrasound, and no history of smoking or alcohol consumption. The exclusion criteria for the PE group included chronic hypertension, diabetes mellitus, heart disease, severe liver or kidney disease, other endocrine disorders, hematologic diseases, recent infection, or substance abuse.

For the control group, 50 healthy pregnant women with singleton mid- to late-stage pregnancies were randomly selected during the same hospitalization period, with no pregnancy-related complications. The exclusion criteria for the control group were severe pregnancy-associated comorbidities or complications. Baseline clinical data, including maternal age, gestational age, blood pressure, and body mass index (BMI), were collected. The study protocol was approved by the Ethics Committee of Hebei Medical University (Approval No. 2024-R581), and written informed consent was obtained from all participants.

#### 2.6.2 Sample collection

Placental tissue samples were collected within 5 min after fetal delivery. Using sterile biopsy forceps, a 1 cm × 1 cm tissue block was excised from the central maternal side of the placenta, avoiding macroscopically visible calcified or infarcted areas. The samples were rinsed with normal saline to remove blood, blotted dry with filter paper, immediately stored in liquid nitrogen, kept for 12 h, and then transferred to a −80 °C freezer for protein extraction and subsequent experiments.

#### 2.6.3 Western blotting

Placental tissue samples were lysed with RIPA buffer (Solarbio, China), and total protein was extracted and quantified. Proteins were separated by SDS-PAGE under low-temperature electrophoresis and then transferred onto PVDF membranes (Millipore, USA) by wet transfer at low temperature for 1 h. After blocking with 5% BSA for 1 h, the membranes were incubated overnight at 4 °C with the following primary antibodies: LEP (1:1,000, ab16227, Abcam), MME (1:1,000, WL02828, Wanleibio), NDRG1 (1:1,000, WL03071, Wanleibio), HK2 (1:1,000, WL02454, Wanleibio), and SERPINA3 (1:1,000, R382127, ZENBIO). After incubation, the membranes were washed and then incubated with Dylight800-conjugated secondary antibody (1:5,000, A23920, Abbine) for 1 h, followed by additional washing. Protein bands were imaged using the Odyssey SLX system (LLX-1215). Finally, band optical density was analyzed using ImageJ software to quantify protein expression levels.

#### 2.6.4 Immunohistochemistry (IHC) staining for spatial cellular localization of five signature genes

To resolve the limitation of bulk Western blot that cannot distinguish cellular origin of upregulated protein expression, immunohistochemical staining was performed on paraffin-embedded placental sections from identical 50 PE and 50 normal control placental samples used for WB validation. Briefly, placental paraffin tissues were routinely fixed in 4% paraformaldehyde, dehydrated, embedded and sliced into 4 μm continuous sections. After antigen retrieval with citrate buffer (pH = 6.0) via high-pressure heating, endogenous peroxidase was blocked by 3% H_2_O_2_, followed by 5% BSA blocking at room temperature for 30 min to eliminate non-specific binding. The same primary antibodies against LEP, HK2, NDRG1, MME and SERPINA3 used for Western blot were incubated overnight at 4 °C with optimized dilution ratios consistent with WB setup. After PBS washing, HRP-conjugated secondary antibody was incubated for 60 min at room temperature, followed by DAB chromogenic reaction and hematoxylin counterstaining. Sections were dehydrated, sealed and observed under light microscopy. The positive staining distribution of each target protein was localized in placental villous trophoblasts (syncytiotrophoblast/cytotrophoblast), decidual stromal cells and placental infiltrating immune cells (macrophage/monocyte/neutrophil). Semi-quantitative integral optical density (IOD) of positive staining was calculated via ImageJ software for statistical comparison between PE and normal groups, with independent-samples t-test for intergroup comparison (P < 0.05 as statistically significant).

### 2.7 Construction, evaluation, and SHAP interpretability analysis of the ANN discriminant model

An artificial neural network (ANN) discriminant model was constructed in R based on the five identified signature genes (LEP, HK2, NDRG1, MME, and SERPINA3). The model consisted of an input layer with 5 nodes, optimized hidden layer configurations determined through cross-validation grid search, and an output layer generating the predicted probability of PE. Training was conducted across the six training cohorts with 5-fold cross-validation, repeated over multiple random seeds (Seeds 42, 52, 62) to assess stability. To address cross-platform technical covariate shifts between the training cohorts and the independent validation cohort (GSE25906), unsupervised feature-level standardization was performed using GSE25906's cohort-specific feature means (μ_val) and standard deviations (σ_val): z = (x - μ_val)/σ_val. Crucially, this harmonization was executed in an entirely unsupervised and label-blinded manner without exposing or utilizing validation outcome labels, ensuring strict cohort independence and zero label leakage. Model performance was comprehensively evaluated using ROC-AUC, accuracy (ACC), sensitivity, specificity, positive predictive value (PPV), negative predictive value (NPV), and F1-score. Confusion matrices were computed to summarize classification outcomes. In addition, SHAP (SHapley Additive exPlanations) values were calculated using the fastshap and shapviz packages to quantify the mathematical predictive contribution of each feature to ANN classifications.

To further interpret the model decision process, SHAP values for each signature gene in the validation cohort were calculated using the fastshap package and visualized with the shapviz package. The predicted probability of the PE class was used as the target for interpretation. A beeswarm plot was used to illustrate the distribution of feature contributions across all samples, while force plots were generated to display the positive or negative contribution of each gene to the prediction of representative individual samples.

### 2.8 Immune infiltration analysis

Immune infiltration analysis was performed on the GSE75010 dataset using the CIBERSORT algorithm based on the LM22 signature matrix. The GSE75010 dataset was selected for immune deconvolution analysis due to its large sample size, well-defined case-control classification, and comprehensive transcriptome profiling. Other training and validation GEO datasets feature relatively small sample sizes or heterogeneous tissue sources; pooling multiple cohorts would introduce substantial batch effects and biological heterogeneity, potentially compromising the stability and reliability of CIBERSORT immune cell deconvolution. Therefore, GSE75010 was prioritized for subsequent immune infiltration evaluations. With 1,000 permutation iterations and quantile normalization, the relative abundances of 22 immune cell types were estimated. Differences in immune cell proportions between groups were compared using the Wilcoxon rank-sum test with Bonferroni/FDR correction, and results were visualized with boxplots and stacked bar charts. Spearman correlation analysis among immune cell types and between signature genes and immune cells was conducted, followed by FDR correction.

### 2.9 Statistical analysis

Statistical analyses were performed using GraphPad Prism 10.1.2 and ImageJ. The Shapiro–Wilk test was used to assess data normality. Normally distributed continuous variables were expressed as mean ± standard deviation (SD), and intergroup comparisons were performed using the independent-samples t-test. Non-normally distributed continuous variables were expressed as medians with quartiles, and intergroup comparisons were performed using the Wilcoxon rank-sum test. A two-sided P < 0.05 was considered statistically significant. Statistical significance was denoted as follows: P < 0.05, P < 0.01, P < 0.001, P < 0.0001, and ns indicated no statistical significance.

## 3 Results

### 3.1 Identification of differentially expressed genes

After merging the GEO datasets GSE10588 and GSE75010, a total of 103 normal samples and 97 preeclampsia (PE) samples were obtained. Principal component analysis (PCA) showed that, before batch correction, samples from GSE10588 and GSE75010 exhibited a clear tendency toward separation in the principal component space (Figure 2A). After ComBat correction, the overlap between samples from the two datasets increased markedly, and batch-related clustering was substantially reduced, indicating that batch effects had been effectively controlled (Figure 2B). Differential expression analysis identified a total of 87 differentially expressed genes (DEGs), including 65 upregulated genes and 22 downregulated genes. All DEGs were visualized using a volcano plot generated with the ggplot2 package (Figure 2C) and a heatmap generated with the pheatmap package (Figure 2D).

**FIGURE 2:**
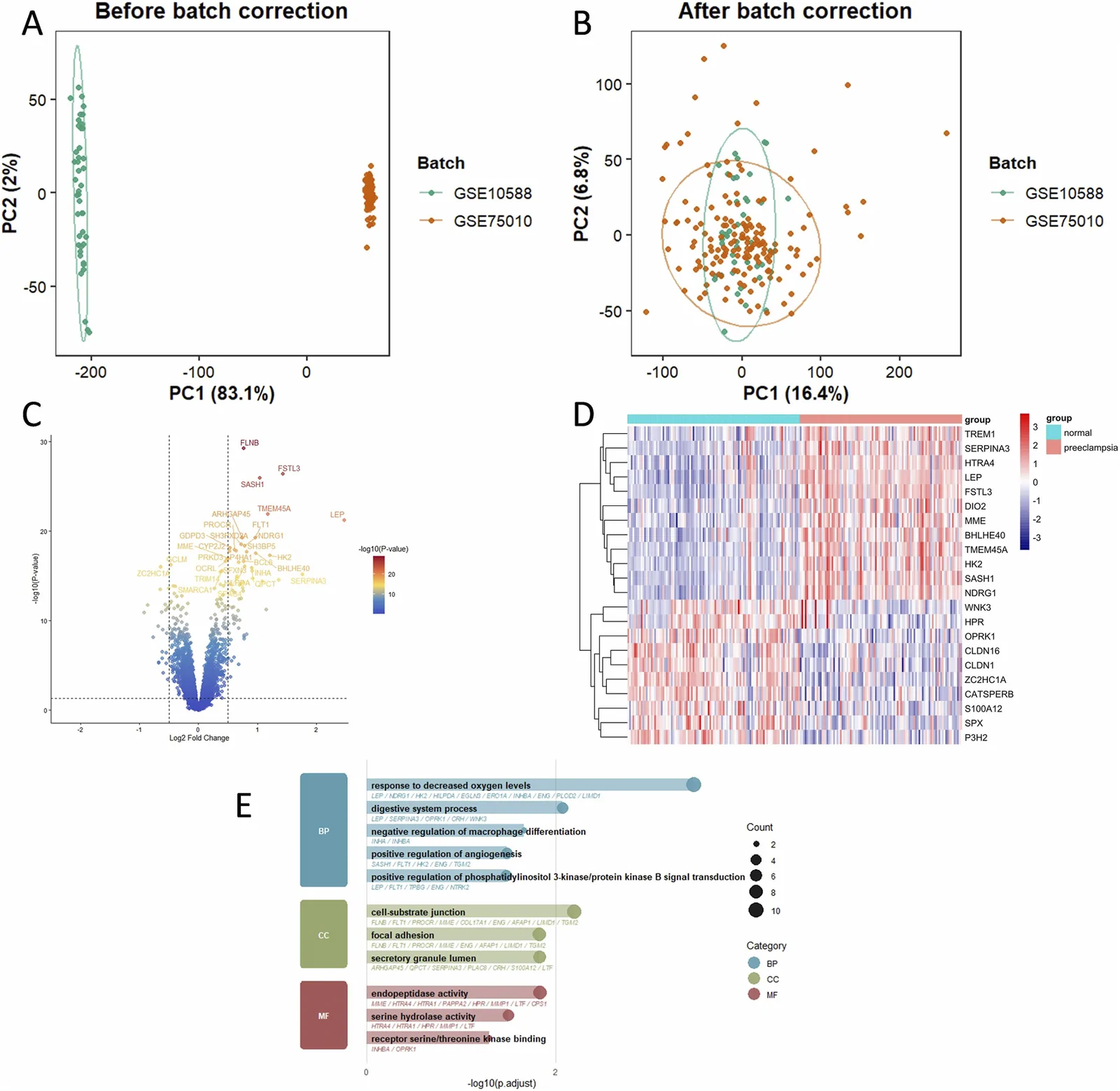
Data preprocessing, differential expression analysis, and functional enrichment of differentially expressed genes (DEGs) in preeclampsia. **(A,B)** Principal component analysis (PCA) before and after batch correction; **(C)** Volcano plot showing upregulated and downregulated DEGs; **(D)** Heatmap depicting gene-expression patterns in the PE and control groups; **(E)** GO functional enrichment analysis.

### 3.2 Functional enrichment and pathway analysis of DEGs

GO enrichment analysis showed that the DEGs were mainly enriched in endocrine process, regulation of gonadotropin secretion, endocrine hormone secretion, inhibin complex, cell-substrate junction, focal adhesion, secretory granule lumen, cytoplasmic vesicle lumen, and functions including monosaccharide binding, L-ascorbic acid binding, peptidyl-prolyl dioxygenase activity, hormone activity, and iron ion binding (Figure 2E).

### 3.3 Screening of oxidative stress-related genes and construction of the PPI network

By intersecting the DEGs with the predefined oxidative stress-related gene set, 17 differentially expressed oxidative stress-related genes (DEOSRGs) were identified (Figure 3A), including LEP, TGM2, BCL6, MME, MMP1, ENG, ABCG2, FLT1, NTRK2, CRH, NDRG1, HK2, ERO1A, LTF, FRZB, SERPINA3, and CP. These DEOSRGs were uploaded to the STRING database for interaction analysis and protein-protein interaction (PPI) network construction, resulting in a PPI network composed of 17 interacting proteins (Figure 3B). In this network, the number of connections between nodes reflects the strength of protein interactions, with more highly connected nodes indicating stronger interaction capacity within the network. The chromosomal localization of these DEOSRGs is shown in Figure 3C.

**FIGURE 3:**
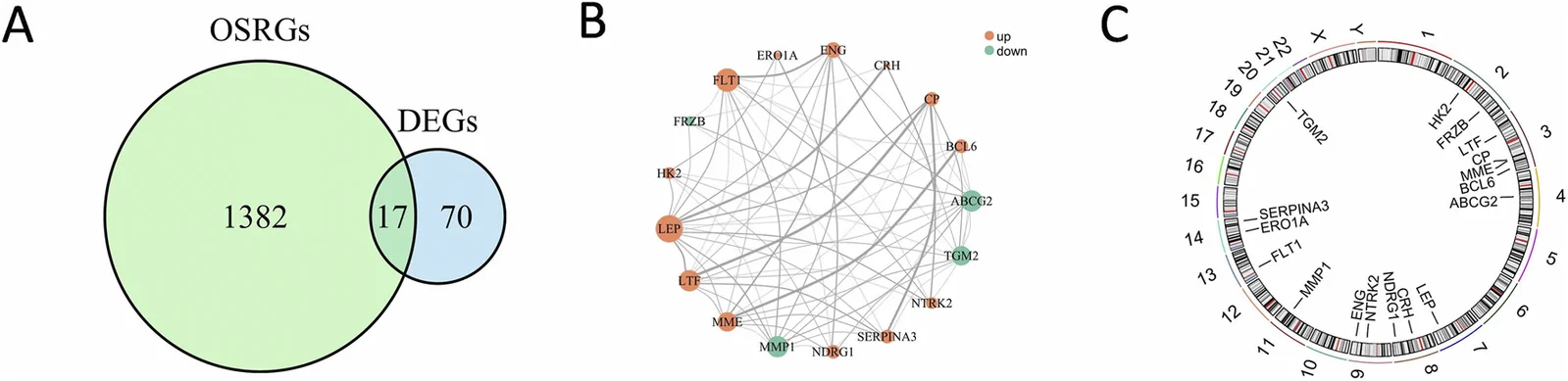
Screening and network analysis of differentially expressed oxidative stress-related genes (DEOSRGs) in preeclampsia. **(A)** Identification of DEOSRGs; **(B)** protein-protein interaction network among DEOSRGs; **(C)** chromosomal localization of DEOSRGs.

### 3.4 Screening and scoring of signature genes

The most discriminative signature genes were identified from the 17 candidate DEOSRGs using SVM-RFE, RF-RFE, and XGB-RFE. Across all three models, 5-fold cross-validation AUC remained stable between 17 and 7 features (maintaining an average AUC of 0.88–0.90, marginal change ΔAUC <0.015). When the feature count was reduced below seven, an accelerated marginal performance loss (ΔAUC >0.045) occurred, indicating that seven features represented the optimal turning point for individual algorithm filtering (Figures 4A,B). Based on the overall importance scores (Figure 4C), the top seven candidate genes were extracted from each algorithm: RF-RFE (TGM2, LEP, HK2, MME, NDRG1, FLT1, SERPINA3), SVM-RFE (LEP, FLT1, NDRG1, HK2, MME, SERPINA3, BCL6), and XGB-RFE (TGM2, LEP, MME, HK2, SERPINA3, NDRG1, ABCG2). By taking the consensus intersection across all three machine learning architectures, five core signature genes were ultimately identified: LEP, HK2, NDRG1, MME, and SERPINA3 (Figure 4D).

**FIGURE 4:**
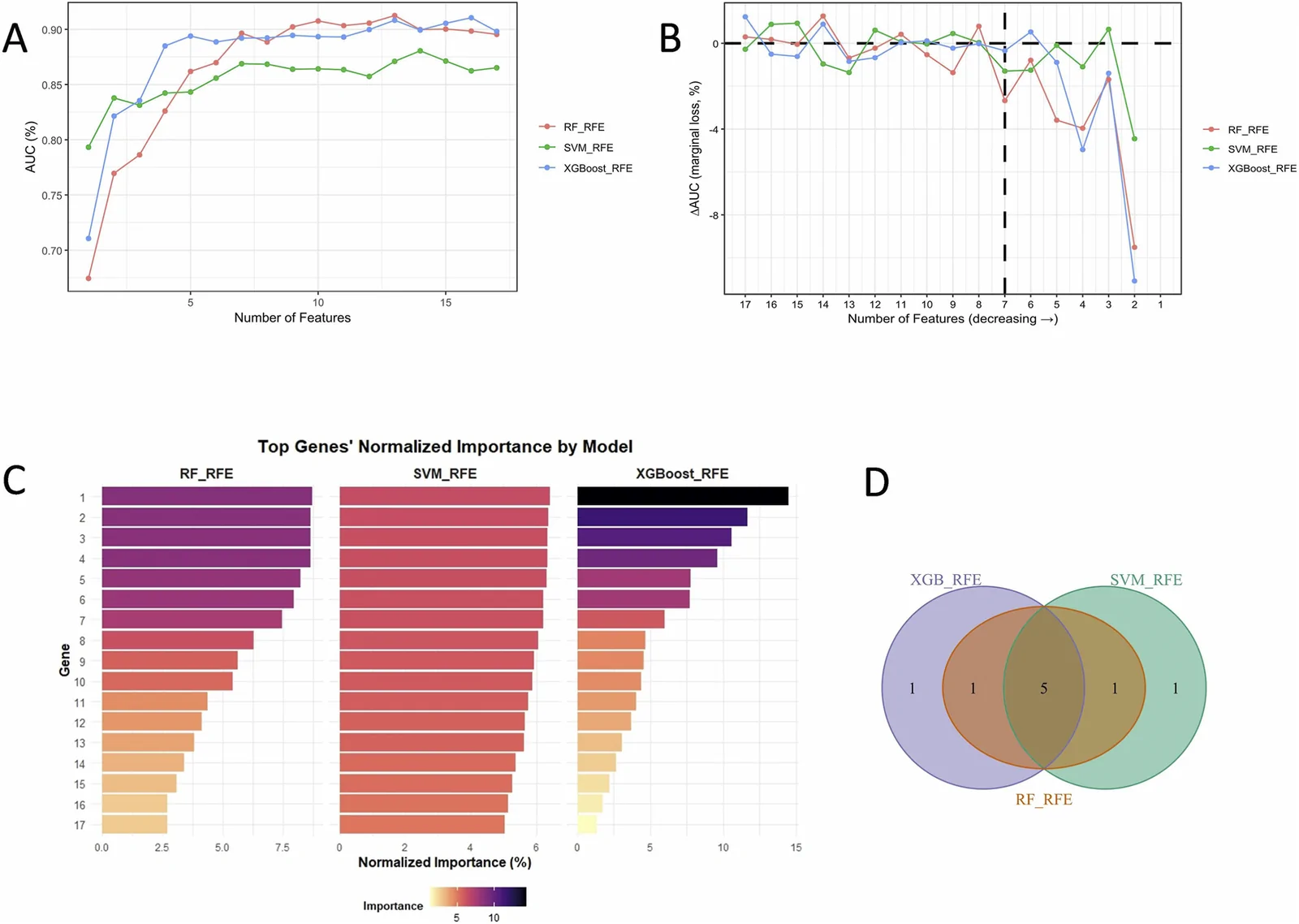
Feature selection and importance evaluation based on multi-algorithm recursive feature elimination (RFE). **(A)** AUC versus feature number curves for SVM-RFE, RF-RFE, and XGB-RFE; **(B)** marginal loss (ΔAUC) during stepwise feature elimination; **(C)** overall importance scores calculated by *varImp* and normalized to 100%; **(D)** intersection of top-ranked genes from different models used to define the consensus signature gene set.

### 3.5 Validation and cellular localization of signature genes in placental tissues

The baseline characteristics of the collected samples are shown in Table 1. Western blotting was performed to validate the expression of the five selected signature genes in placental tissues from patients with PE (Figures 5A,B). The expression levels of LEP, MME, NDRG1, HK2, and SERPINA3 were all significantly higher in the PE group than in the normal control group (P < 0.05).

**TABLE 1:** Baseline characteristics.

Category	Clinical characteristic	Control (n = 50)	PE (n = 50)	t/Z	*p*
​	Age (years)	31.82 ± 6.124	32.66 ± 4.716	−0.768	0.444
​	Gestational week (weeks)	35.38 ± 2.890	33.34 ± 2.967	3.475	<0.001
Maternal	Gravidity	2 (1,3.25)	2 (1,3)	0.398	0.691
​	Parity	0.5 (0,1)	0 (0,1)	0.601	0.549
​	Gestational weight gain (kg)	10.91 ± 5.580	13.61 ± 5.456	−2.116	0.038
​	SBP(mmHg)	114.94 ± 19.026	145.96 ± 17.372	−8.475	<0.001
​	DBP(mmHg)	76.61 ± 10.460	99.32 ± 11.329	−10.356	<0.001
​	Birth weight (g)	2,539.20 ± 752.389	1846.60 ± 808.273	4.435	<0.001
​	Apgar 1 min	8 (8,9)	8 (7,8)	3.707	<0.001
Neonatal	Apgar 5 min	9 (9,10)	9 (8,9)	3.652	<0.001
​	Apgar 10 min	9.5 (9,10)	9 (8,9)	3.399	0.001

**FIGURE 5:**
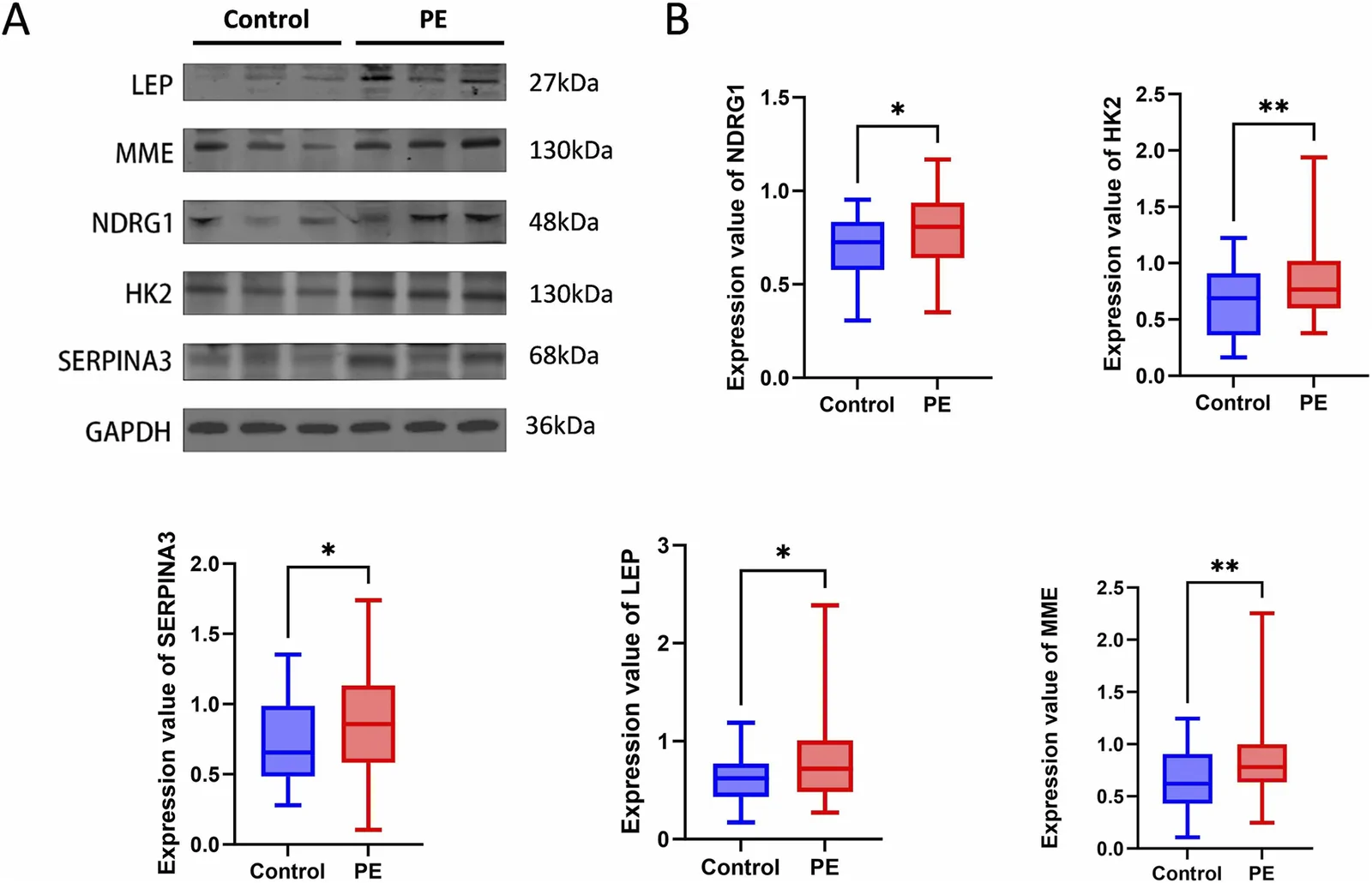
Validation of oxidative stress signature genes in placental tissues from 50 preeclampsia cases and 50 controls. **(A)** Western blot (WB) detection of the protein levels of five oxidative stress signature genes; **(B)** Visualization of protein expression levels for the signature genes. (*P < 0.05, **P < 0.01).

To further address the limitation that bulk placental Western blotting cannot distinguish the cellular source of protein expression, and to clarify the spatial localization of the five signature proteins within the heterogeneous placental tissue, immunohistochemical staining was additionally performed to examine the *in situ* expression distribution of LEP, HK2, NDRG1, MME, and SERPINA3 in placental tissues (Figure 6). The results showed that positive signals for LEP, HK2, NDRG1, MME, and SERPINA3 were mainly distributed in placental villous trophoblasts, particularly in the syncytiotrophoblast region, suggesting that the increased protein expression detected by Western blotting was mainly derived from enhanced expression in trophoblast cells. In addition, weak positive staining was observed in some flattened or spindle-shaped cells surrounding villous blood vessels, suggesting possible expression in vascular endothelial-related cells. Scattered round or oval positive cells were also observed within the villous stroma, indicating that stromal cells or immune-like cell components may contribute to local positive signals. Collectively, these IHC results indicate that the increased expression of the five signature proteins in PE placental tissues is mainly associated with enhanced positive expression in the villous trophoblast layer, especially in the syncytiotrophoblast region, while vascular endothelial-related cells and villous stromal/immune-like cells may also participate in local expression changes.

**FIGURE 6:**
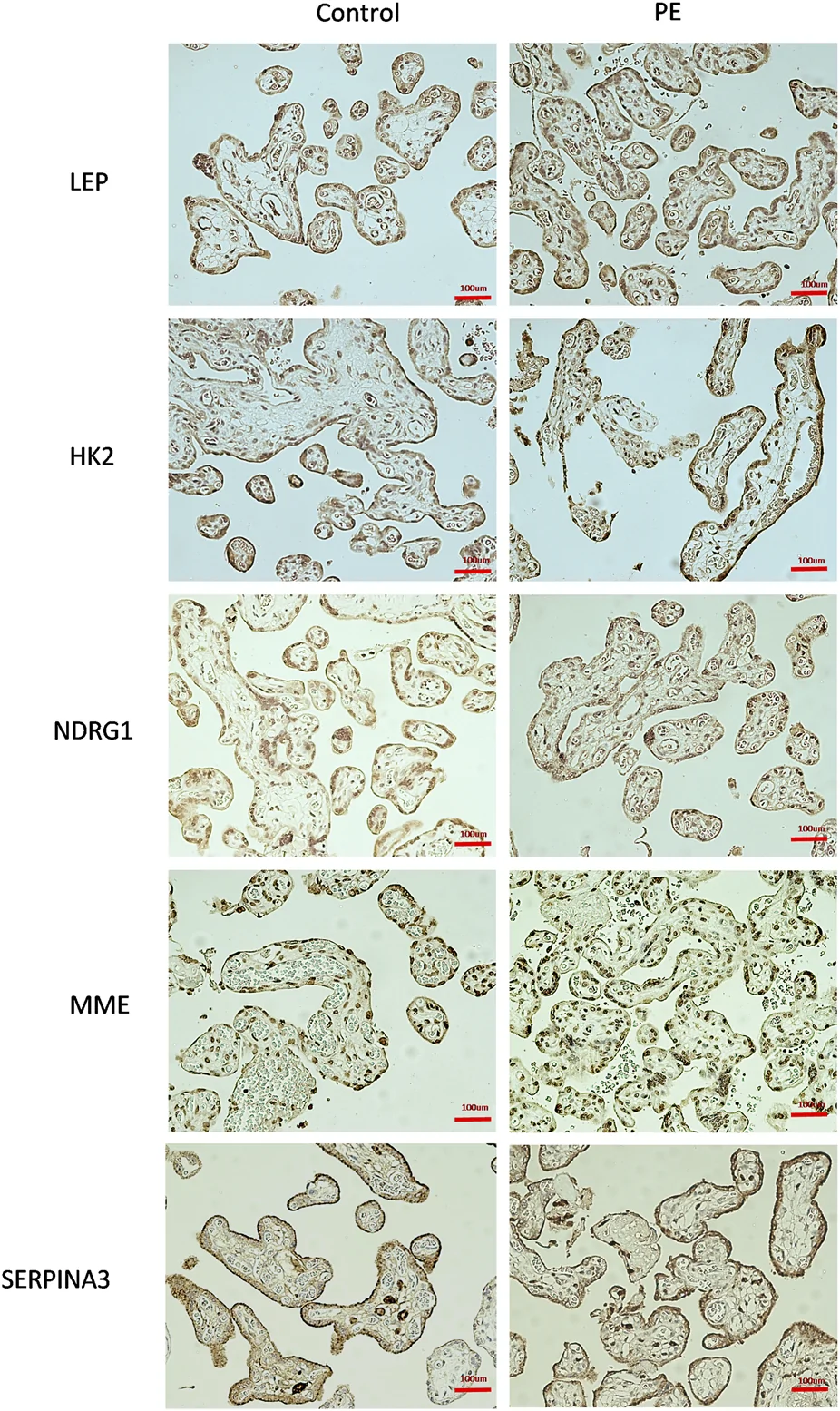
Immunohistochemical localization of five signature proteins in normal and preeclampsia placental tissues.

### 3.6 Construction, evaluation, and SHAP interpretability analysis of the ANN discriminant model

Based on the five signature genes (LEP, HK2, MME, NDRG1, and SERPINA3), an artificial neural network (ANN) discriminant model was constructed. Boxplots confirmed significant differential expression of all five genes between PE and normal controls in the independent validation dataset GSE25906 (Figure 7A). The ANN model architecture is illustrated in Figure 7B. In 5-fold cross-validation across the training cohorts, the ANN model achieved an average AUC of 0.888 ± 0.010 (accuracy: 81.5%, sensitivity: 83.0%, specificity: 79.7%, PPV: 83.9%, NPV: 78.6%, F1-score: 0.834) across three independent random seeds (Figure 7C). When tested on the completely independent external validation cohort GSE25906 (N = 60; 23 PE vs. 37 controls), the model yielded a robust AUC of 0.819 ± 0.010, an accuracy of 73.3%, a sensitivity of 73.9%, a specificity of 73.0%, a PPV of 63.0%, an NPV of 81.9%, and an F1-score of 0.680 (confusion matrix: 17 true positives, 6 false negatives, 27 true negatives, 10 false positives).

**FIGURE 7:**
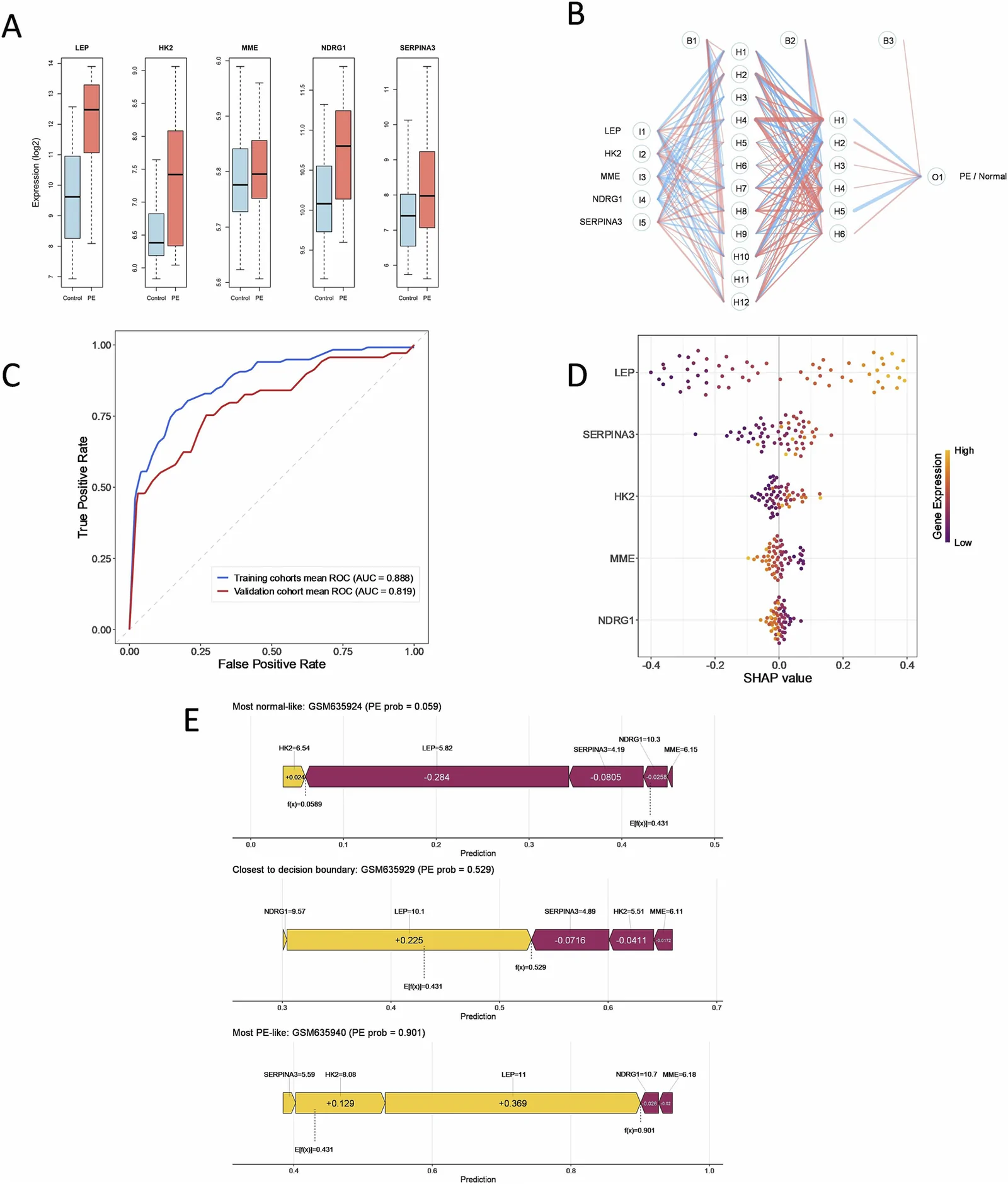
Performance visualization and interpretation of the artificial neural network (ANN) model. **(A)** Boxplots of the five feature genes in the GSE25906 validation cohort; **(B)** Schematic of a two-hidden-layer ANN with a 5-12-6-1 architecture; **(C)** ROC curves of the training cohorts (mean AUC = 0.888 ± 0.010 across three random seeds) and the GSE25906 validation cohort (mean AUC = 0.819 ± 0.010); **(D)** SHAP beeswarm plot showing feature contributions in the GSE25906 validation cohort; **(E)** SHAP force plot illustrating individual-level prediction paths in the GSE25906 validation cohort.

Crucially, to verify whether the final 5-gene consensus signature achieves performance comparable to the 7-gene panels, head-to-head quantitative comparisons were evaluated on the independent validation cohort GSE25906: the 7-Gene SVM model yielded an AUC of 0.816 (accuracy 71.7%), the 7-Gene RF model yielded an AUC of 0.821 (accuracy 73.3%), the 7-Gene XGBoost model yielded an AUC of 0.814 (accuracy 71.7%), and the final 5-Gene consensus ANN model achieved an AUC of 0.819 ± 0.010 (accuracy 73.3%, sensitivity 73.9%, specificity 73.0%). These results demonstrate that the 5-gene consensus signature effectively captures the key discriminatory information of the 7-gene panels, simplifying model complexity while maintaining equivalent discriminative performance. Furthermore, SHAP values were calculated to evaluate the predictive contribution of each feature to the ANN prediction output. A SHAP beeswarm plot showed that LEP and MME exerted the greatest predictive impact on classification, followed by HK2, SERPINA3, and NDRG1 (Figure 7D). SHAP force plots of representative samples illustrated the feature attributions for individual predictions (Figure 7E). Importantly, high SHAP attribution reflects algorithmic feature importance within the trained model rather than biological causality in disease etiology.

### 3.7 Results of immune infiltration analysis

Immune infiltration analysis estimated the overall distribution of 22 immune cell types in placental tissues from the normal pregnancy group and the PE group in the GSE75010 dataset (Figure 8A). In both groups, the placental immune microenvironment was mainly composed of monocytes, macrophages, T cells, and NK cells. Further comparison between groups (Figure 8B) showed that resting mast cells were significantly increased in the PE group, whereas M2 macrophages, monocytes, neutrophils, and plasma cells were significantly decreased. NK cells did not show statistically significant changes. These findings suggest that immune dysregulation and inflammatory responses may occur in patients with PE.

**FIGURE 8:**
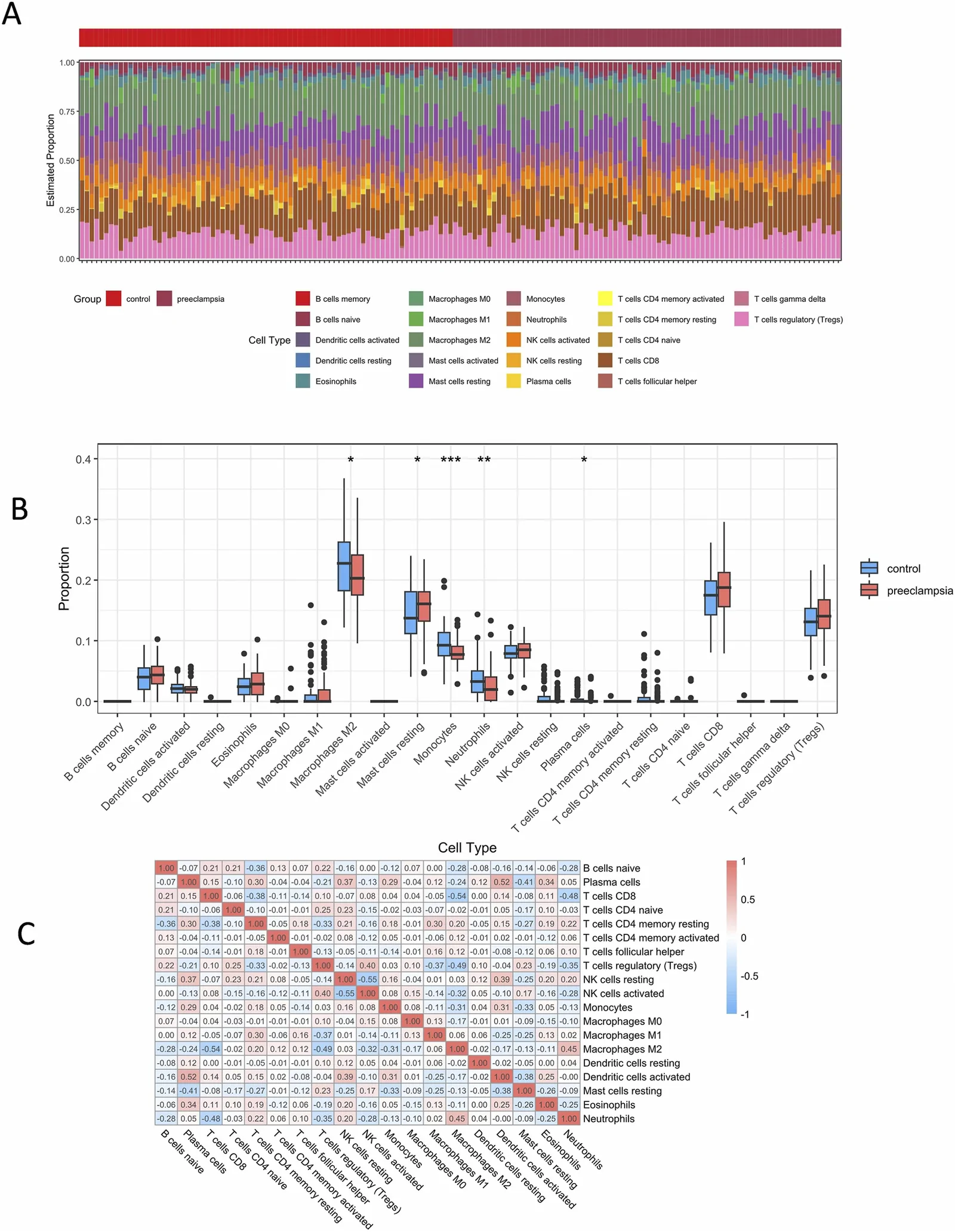
Immune infiltration analysis and visualization using CIBERSORT in GSE75010. **(A)** Stacked composition of immune cell subsets in normal vs. preeclampsia groups; **(B)** Boxplots visualizing between-group differences in cell proportions; **(C)** Correlation heatmaps of immune cells in normal and preeclampsia groups. *P < 0.05, **P < 0.01 ***P < 0.001.

The cell-cell correlation matrix (Figure 8C) showed that M2 macrophages were negatively correlated with CD8 T cells and Tregs (correlation coefficients: −0.54 and −0.49, respectively), whereas plasma cells were positively correlated with activated dendritic cells (0.52), indicating that coordinated alterations among these immune cell populations may contribute to the development of PE.

We further analyzed the correlations between the five candidate genes and immune cell infiltration (Figure 9). The results showed that these genes were mainly associated with monocytes, M2 macrophages, neutrophils, and Treg cells. LEP, HK2, and NDRG1 were positively correlated with Tregs and resting mast cells, while showing significant negative correlations with monocytes, neutrophils, and M2 macrophages. MME was positively correlated with eosinophils and significantly negatively correlated with M2 macrophages. SERPINA3 was mainly characterized by negative correlations with monocytes and neutrophils. These findings suggest that these key genes may participate in the regulation of autoimmunity through modulation of immune cell expression.

**FIGURE 9:**
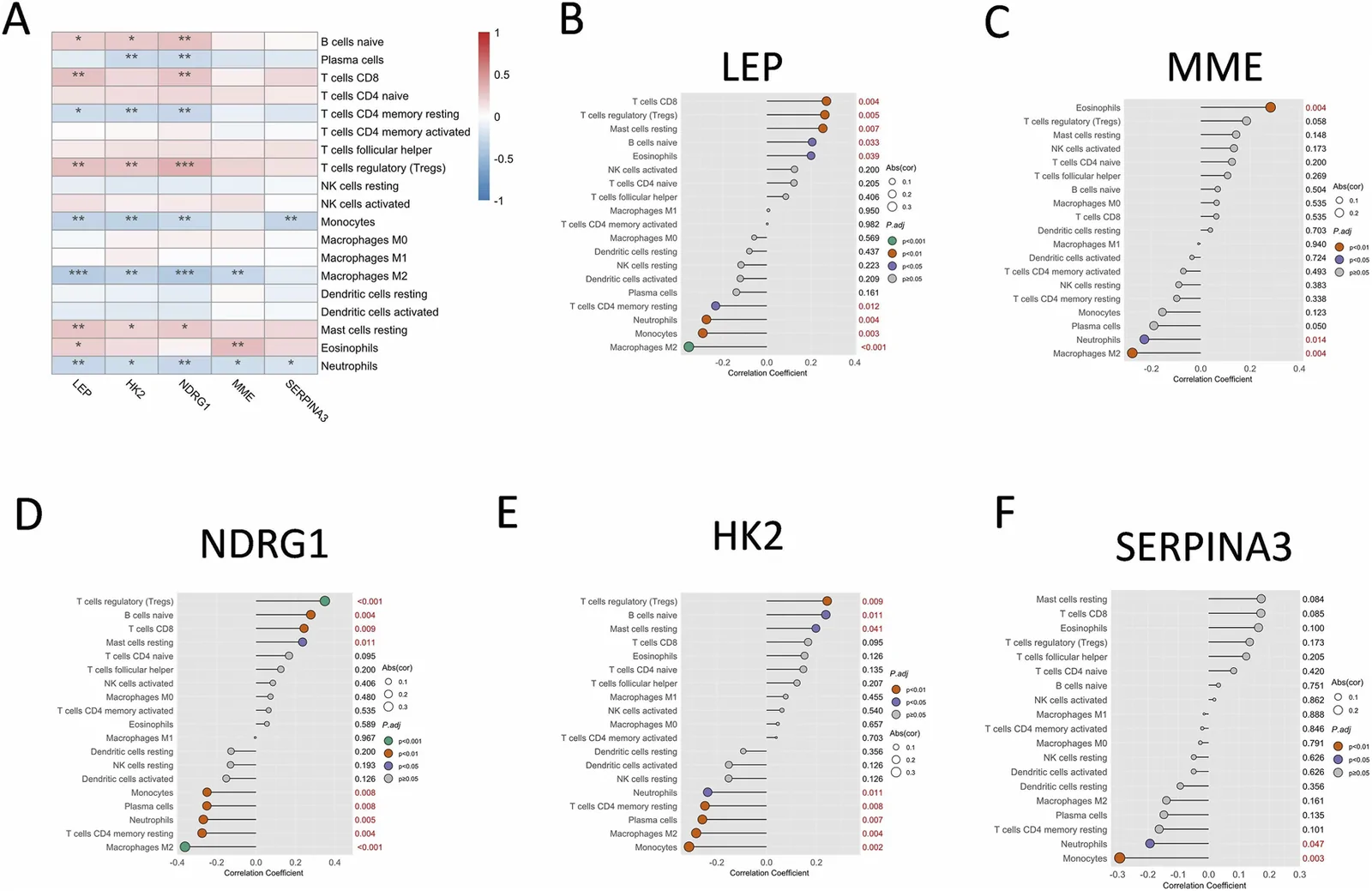
Correlation analysis and visualization of feature genes with immune cells. **(A)** Immune infiltration analysis of 5 feature genes. **(B–F)** Correlation analysis between feature genes and immune cells.

## 4 Discussion

The pathogenesis of preeclampsia (PE) is highly complex, and candidate biomarkers for risk stratification and molecular characterization remain urgently needed. In this study, we established a systematic multi-algorithm machine learning framework to identify five core oxidative stress-related signature genes (LEP, HK2, NDRG1, MME, and SERPINA3) from transcriptomic datasets, validated their protein expression and trophoblast localization in clinical placental specimens, constructed an interpretable ANN discriminant model with robust external performance (AUC = 0.819 in GSE25906), and elucidated their close correlations with the placental immune microenvironment.

Leptin (LEP), as an important adipokine and placental hormone, appears to play a central role in the pathogenesis of PE. Its elevated expression in the serum and placental tissues of patients with PE has been consistently reported in multiple studies (Brew et al., 2016; Laivuori et al., 2006; El et al., 2013; Molvarec et al., 2011). In the present study, this phenomenon was further confirmed by both bioinformatics analysis and Western blotting. Moreover, SHAP analysis was employed to provide an interpretable representation of the ANN prediction process, thereby characterizing the direction and relative magnitude of the contribution of different signature genes in the validation cohort. These findings further support the plausibility of LEP as a candidate biomarker for PE. Mechanistically, LEP may amplify pro-inflammatory responses and oxidative stress by activating signaling pathways such as JAK-STAT and PI3K/Akt, thereby inducing endothelial dysfunction. Accordingly, LEP is not only a candidate molecular marker, but may also represent a key molecule linking metabolic abnormalities with the oxidative stress–inflammation network in PE (Mellott and Faulkner, 2023). From the perspective of immune regulation, the immune infiltration results of this study showed a positive correlation between LEP and Treg cells. Taken together with the pro-inflammatory and oxidative stress-promoting effects of LEP, it may be inferred that LEP expression is correlated with local placental immune tolerance by modulating the differentiation and function of Treg cells through enhanced oxidative stress. This inference is also consistent with the central pathological framework of “oxidative stress–immune imbalance” in PE and adds an immunoregulatory dimension to the potential mechanistic network linking altered LEP expression to disease progression.

Hexokinase 2 (HK2), a key rate-limiting enzyme in glycolysis (Guo et al., 2022), appears to play a complex role in PE. Conventional views suggest that glycolytic pathways are globally suppressed in PE, accompanied by reduced cellular energy status (Bloxam et al., 1987), and that HK2 is downregulated in decidual tissue, where it impairs endometrial stromal cell function (Lv et al., 2018). However, in the present study, machine learning-based screening identified HK2 as a key upregulated signature gene in placental tissue. This seemingly contradictory observation suggests the existence of tissue-specific and context-dependent regulation of HK2 in PE. Such regulation may involve multiple levels. At the post-transcriptional level, the RNA-binding protein SORBS2 can promote trophoblast proliferation and migration by stabilizing HK2 mRNA (Song et al., 2024) At the epigenetic level, HK2P1 (the HK2 pseudogene) can upregulate HK2 expression by competitively sponging miR-6887-3p, thereby affecting decidualization (Lv et al., 2018) More importantly, the present study directly links HK2 to oxidative stress, a core pathological feature of PE. Recent mechanistic evidence indicates that the oxidative stress mediator H_2_O_2_ can directly inhibit lactylation at the 346th site of HK2, a modification critical for its mitochondrial binding, glycolytic activity, and cell proliferation (Chen et al., 2025). This provides strong molecular support for why HK2 was captured by the oxidative stress-oriented gene screening strategy used in this study. In the PE microenvironment, HK2 is likely not merely a participant in metabolic alterations, but also a direct target of oxidative stress, with its dysfunction mediating oxidative stress-induced trophoblast injury. Thus, the identification of HK2 as a signature gene highlights its central position at the intersection of metabolic reprogramming and oxidative stress. Furthermore, immune infiltration analysis showed a significant negative correlation between HK2 and M2 macrophages, which are known to promote tissue repair and placental vascular remodeling (Yao et al., 2019). This finding suggests that altered HK2 expression under oxidative stress is inversely correlated with M2 macrophage infiltration, and this correlation may be linked to defective placental vascular remodeling and extending the mechanistic chain linking oxidative stress to immune cell dysregulation in PE.

The role of membrane metallo-endopeptidase (MME) in the pathogenesis of PE is receiving increasing attention. Although its direct association with oxidative stress remains to be fully clarified, multiple studies have suggested that MME is aberrantly expressed in PE placental tissues and may participate in disease progression by regulating the balance of vasoactive peptides. In one study employing two-stage feature selection combined with a convolutional neural network, MME was consistently identified as a core gene across multiple datasets and was further classified as one of the few nodes strongly associated with PE in a molecular regulatory network, highlighting its potential regulatory value at the systems level (Sarker et al., 2025). Another independent experimental study confirmed significant upregulation of MME at both the protein and mRNA levels in PE placental tissues using immunohistochemistry and quantitative PCR (Jiang et al., 2022), in agreement with the Western blot results of the present study. Notably, oxidative stress in the pathological environment of PE may indirectly regulate MME expression by affecting transcription factor activity or epigenetic modification, whereas MME, as a membrane metalloendopeptidase, may itself participate in oxidative stress-related pathways through the hydrolysis of bioactive peptides. Collectively, these findings indicate that MME is not only a candidate biomarker for PE, but may also represent an important link between oxidative stress and the clinical manifestations of the disease. In the present study, immune infiltration analysis showed that MME was significantly negatively correlated with M2 macrophages and positively correlated with eosinophils. Given the enzymatic function of MME, it may be speculated that oxidative stress-associated elevated MME expression correlates with changes in placental immune microenvironment, possibly via the hydrolysis of bioactive peptides that relate to M2 macrophage and eosinophil activity.

NDRG1, a typical hypoxia-responsive stress molecule, has been shown to be specifically upregulated in PE placental tissues, and its expression is strongly induced by hypoxic and oxidative stress signals (Shi et al., 2013). In the present study, multiple machine learning algorithms precisely identified NDRG1 from stress-related genes, and the results demonstrated its good discriminative value, in agreement with previous reports. Mechanistically, the functional effects of NDRG1 appear to be systemic. It can impair trophoblast invasion by inhibiting the ERK/MMP-9 pathway and suppress angiogenesis by modulating the balance among VEGF, PLGF, and sFlt-1 (Dai et al., 2020; Fu et al., 2017). In addition, NDRG1 may inhibit cell proliferation and accelerate cellular senescence, thereby exacerbating placental dysfunction through multiple dimensions (Zhang et al., 2025; Wu and Zhao, 2025). These findings establish a coherent logic chain extending from “oxidative stress-induced expression,” to “pathology mediated by cellular dysfunction,” and further to “biomarker potential reflected by discriminative performance,” thereby elevating NDRG1 from a background stress indicator to a key biomarker with both mechanistic significance and translational potential. In combination with the immune infiltration results, NDRG1 was positively correlated with Treg cells and negatively correlated with M2 macrophages. Since abnormal Treg expansion and impaired M2 macrophage function can both disrupt placental immune tolerance and vascular remodeling (Lindau et al., 2021), these findings suggest that altered NDRG1 expression under oxidative stress correlates with the infiltration of Treg cells and M2 macrophages, and such correlations may be associated with aggravated placental pathological injury. This additional mechanistic perspective makes the role of NDRG1 in PE more biologically comprehensive.

As a member of the serine protease inhibitor family, SERPINA3 shows consistently elevated expression in PE placental tissues and may represent a tissue-specific biomarker candidate (Pinto-Souza et al., 2025; Doridot et al., 2014). Existing studies have revealed multilayered regulatory mechanisms underlying its dysregulation in PE. At the epigenetic level, hypomethylation of CpG islands in the promoter region, together with specific SNP loci, jointly drives transcriptional activation of SERPINA3 (Chelbi et al., 2007). At the transcriptional level, hypoxic signaling induces its expression through an HIF-1-mediated transcription factor network involving SP1, MZF1, and others, whereas persistent oxidative stress may reinforce its high-expression pattern by maintaining an abnormal methylation state. Functional studies have shown that overexpression of SERPINA3 significantly impairs trophoblast adhesion and delays apoptosis (Chelbi et al., 2012), thereby providing mechanistic clues for abnormal placental remodeling in PE. More importantly, bioinformatics analyses have shown that SERPINA3 is significantly associated with the infiltration of plasma cells, eosinophils, and multiple activated immune cell populations (Yang et al., 2022), suggesting that its aberrant expression is associated with PE progression alongside changes in the immune microenvironment. In the present study, machine learning identified SERPINA3 as an oxidative stress-related signature gene, which not only supports its discriminative value in PE, but also places it within a novel mechanistic framework of “oxidative stress–epigenetic regulation–immune modulation,” providing an important direction for future investigation of its complete functional pathway in PE. Specifically, immune infiltration analysis in this study showed that SERPINA3 was negatively correlated with monocytes and neutrophils. Since monocyte dysfunction and excessive neutrophil activation are both closely associated with inflammatory responses in PE, it may be inferred that sustained high expression of SERPINA3, maintained by oxidative stress, altered SERPINA3 expression correlates with inflammatory status in PE, alongside variations in monocyte differentiation and neutrophil activation.

Taken together, the five oxidative stress-related signature genes identified in this study (LEP, HK2, NDRG1, MME, and SERPINA3) do not function in isolation. Rather, their altered expression reflects coordinated stress pathways linked to hypoxia and metabolic adaptations. Under oxidative stress, these genes correlate with immune cell remodeling (Tregs, M2 macrophages, monocytes) through metabolic reprogramming (HK2), inflammatory amplification (LEP), cellular functional regulation (NDRG1, MME), and protease balance (SERPINA3). This integrated network provides a biological rationale for the discriminative power of the five-gene panel.

The present study has several strengths. First, three machine learning-based recursive feature elimination algorithms were jointly applied during the feature screening stage, ultimately yielding a five-gene signature panel that balanced parsimony with classification performance. Second, six independent training cohorts were included to construct the ANN model, and an additional independent dataset was used for external validation, thereby improving the robustness and applicability of the model across datasets to some extent. Third, Western blotting of placental tissues confirmed expression changes of the key genes at the protein level, while SHAP analysis further clarified the direction and relative magnitude of the contribution of each signature gene to ANN predictions, thus enhancing the credibility of the findings from both experimental and modeling perspectives. Fourth, immune infiltration analysis was incorporated to explore the potential relationship between the signature genes and the placental immune microenvironment, which endows the present findings not only with discriminative value but also with preliminary biological relevance.

Several limitations of the present study should be acknowledged. First, because the predictive model was constructed using transcriptomic and tissue measurements derived from postpartum placental specimens, it characterizes local tissue-level molecular alterations rather than serving as an antepartum non-invasive screening tool; prospective validation in maternal peripheral circulation is required before clinical translational utility can be established. Second, in retrospective public microarray cohorts, gestational age and clinical subtype reporting (early-onset vs. late-onset PE) exhibit inherent variations, which represent potential clinical confounders. Third, immune infiltration analysis was primarily conducted in single-platform cohort; future multi-center studies with single-cell RNA sequencing and prospectively matched gestational age cohorts will be essential to further resolve cellular dynamics.

In future studies, we will further expand multi-dataset immune validation, establish prospective cohorts with better-matched clinical covariates, and perform mechanistic cellular and animal experiments to clarify the functional roles of these genes. In addition, given that the current model is based on postpartum placental tissue, future work will explore the potential of these signature genes as non-invasive peripheral blood biomarkers for early prenatal prediction of preeclampsia. Overall, these findings deepen our understanding of PE and provide a basis for candidate biomarker development and further mechanistic investigation.

## Data Availability

The datasets presented in this study can be found in online repositories. The names of the repository/repositories and accession number(s) can be found in the article/Supplementary Material.
